# Fast Gamma Rhythms in the Hippocampus Promote Encoding of Novel Object–Place Pairings

**DOI:** 10.1523/ENEURO.0001-16.2016

**Published:** 2016-05-12

**Authors:** Chenguang Zheng, Kevin Wood Bieri, Ernie Hwaun, Laura Lee Colgin

**Affiliations:** 1 Center for Learning and Memory, University of Texas at Austin, Austin, Texas 78712-0805; 2 Department of Neuroscience, University of Texas at Austin, Austin, Texas 78712-0805; 3 Institute for Neuroscience, University of Texas at Austin, Austin, Texas 78712-0805

**Keywords:** CA1, gamma oscillations, gamma rhythms, hippocampus, memory, place cells

## Abstract

Hippocampal gamma rhythms increase during mnemonic operations (Johnson and Redish, 2007; Montgomery and Buzsáki, 2007; Sederberg et al., 2007; Jutras et al., 2009; Trimper et al., 2014) and may affect memory encoding by coordinating activity of neurons that code related information (Jensen and Lisman, 2005). Here, a hippocampal-dependent, object–place association task (Clark et al., 2000; Broadbent et al., 2004; Eacott and Norman, 2004; Lee et al., 2005; Winters et al., 2008; Barker and Warburton, 2011) was used in rats to investigate how slow and fast gamma rhythms in the hippocampus relate to encoding of memories for novel object–place associations. In novel object tasks, the degree of hippocampal dependence has been reported to vary depending on the type of novelty (Eichenbaum et al., 2007; Winters et al., 2008). Therefore, gamma activity was examined during three novelty conditions: a novel object presented in a location where a familiar object had been (NO), a familiar object presented in a location where no object had been (NL), and a novel object presented in a location where no object had been (NO+NL). The strongest and most consistent effects were observed for fast gamma rhythms during the NO+NL condition. Fast gamma power, CA3–CA1 phase synchrony, and phase-locking of place cell spikes increased during exploration of novel, compared to familiar, object–place associations. Additionally, place cell spiking during exploration of novel object–place pairings was increased when fast gamma rhythms were present. These results suggest that fast gamma rhythms promote encoding of memories for novel object–place associations.

## Significance Statement

This study provides the first evidence that links fast gamma rhythms in the hippocampus to encoding of novel object–place associations in a behavioral task. The results also relate these effects to firing patterns in place cells that resemble stimulation patterns that are routinely used to induce long-term potentiation, the presumed synaptic substrate of memory formation.

## Introduction

Gamma oscillations (∼25–100 Hz) are prominent in the entorhinal-hippocampal network and have been shown to appear during a variety of memory tasks in rats, monkeys, and humans (Fell et al., 2001; Johnson and Redish, 2007; Montgomery and Buzsáki, 2007; Sederberg et al., 2007; Jutras et al., 2009; Trimper et al., 2014). Gamma rhythms occur as two distinct variants that are thought to route different streams of information entering hippocampal subfield CA1 (Colgin et al., 2009; Schomburg et al., 2014). Slow gamma (∼25–55 Hz) may facilitate transmission of inputs to CA1 from CA3, a hippocampal subfield thought to be important for memory retrieval (Sutherland et al., 1983; Brun et al., 2002; Steffenach et al., 2002). Fast gamma (∼60–100 Hz) may promote inputs from the medial entorhinal cortex (MEC) that transmit ongoing spatial information (Brun et al., 2002; Fyhn et al., 2004; Hafting et al., 2005). Functional correlates of these gamma subtypes have been reported for CA1 place cells in the form of different spatial coding modes (Bieri et al., 2014; Zheng et al., 2016). The firing properties exhibited in each case were hypothesized to reflect cellular mechanisms of memory retrieval during slow gamma and memory encoding during fast gamma. However, if these neuronal coding modes are involved in memory function, then effects should also be evident during behaviors in which these mnemonic processes are explicitly demonstrated. In the present study, memory encoding and retrieval were examined at the behavioral level using an object–place association task. Slow and fast gamma activities were measured during periods of exploration of novel and familiar object–place pairings. Memory encoding presumably occurs during exploration of novel object–place pairings, and memory retrieval presumably occurs during exploration of familiar object–place pairings.

In standard novel object exploration tasks, rats are presented with a novel object and a familiar object in the same environment and are allowed to freely explore each item. Rats have been shown to spend more time exploring novel objects compared with familiar objects (Ennaceur and Delacour, 1988), providing behavioral evidence that rats recognize one object as novel and the other as familiar. The ability to discriminate novel and familiar objects is impaired in rats with hippocampal lesions, but this deficit is variable and appears to depend on the specific type of novelty involved (Eichenbaum et al., 2007; Winters et al., 2008). When novelty involves only the identity of the object, some studies report no deficits in rats with hippocampal lesions (Mumby et al., 2002; Winters et al., 2004), whereas other studies report variable deficits depending on the size of the lesion (Broadbent et al., 2004) or the length of delay between familiarization and novelty exposure (Clark et al., 2000). In contrast, when novelty involves changes in the location of an object, deficits are more reliably observed following hippocampal lesions (Eacott and Norman, 2004; Lee et al., 2005; Winters et al., 2008; Barker and Warburton, 2011).

**Figure 1.**
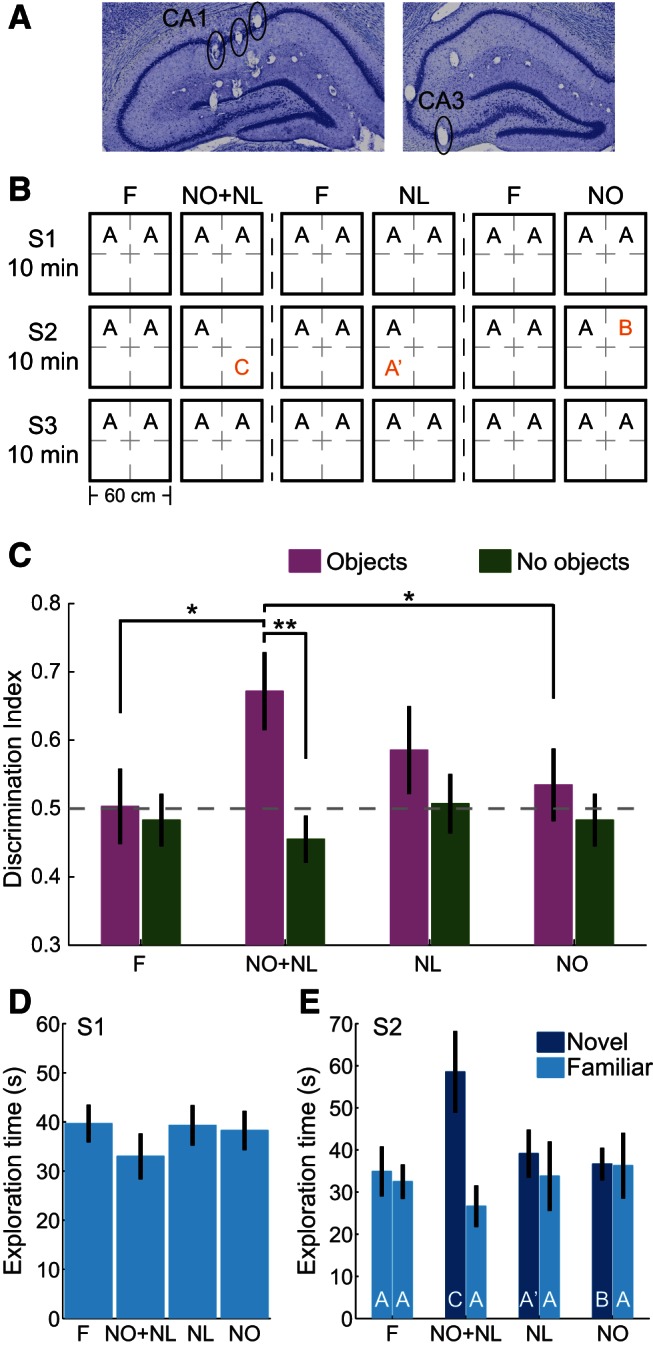
Verification of target recording sites and behavioral effects in object–place association task. ***A***, Histologic sections showing example recording sites in CA1 and CA3. ***B***, A schematic explaining the object–place association task is shown. The behavioral task consisted of 3 familiarization days (F; Object A) and 3 d in which novel object–place pairings were presented. The novel-object place pairings included an object identity and location change (NO+NL; Object C), a location change only (NL; Object A′), and an object identity change only (NO; Object B). Each day consisted of three 10 min exploration sessions (S1, S2, S3) separated by 10 min rest periods, and the order of the conditions was randomly assigned for each animal. ***C***, The discrimination index for the familiarization and novelty conditions, as well as control conditions in which no objects were presented. Grey dashed line indicates chance level. For NO+NL conditions, rats explored the novel object–place pairings significantly more than the familiar object–place pairings and significantly more than they explored the same locations when no objects were present. Because novel object–place pairings were presented in the second session, familiarization measures were also computed using the second session of familiarization or re-familiarization days (F) in this figure and all subsequent figures. ***D***, The amount of time rats spent exploring familiar object–place pairings in Session 1 (S1) of the familiarization condition and the different novelty conditions. ***E***, The amount of time rats spent exploring familiar (light blue bars; Object A indicated in white text) and novel (dark blue bars; Objects C, A′, and B indicated in white text) object–place pairings in Session 2 (S2) of the familiarization condition and the different novelty conditions. *p < 0.05, **p < 0.01. Data are presented as mean ± SEM in this figure and all subsequent figures.

Due to the reported variability of hippocampal involvement in novel object exploration tasks, gamma activity was examined during three types of novelty: novel object identity (NO), novel object location (NL), and novel object identity in a novel object location (NO+NL; Fig. 1*B*). When both object identity and location were changed (ie, NO+NL), behavioral effects of novelty were observed, and fast gamma measures were consistently heightened when animals explored the novel object–place pairings. Moreover, in the NO+NL condition, CA1 place cell firing rates increased selectively during periods of fast gamma, and place cell spikes were strongly phase-locked to fast gamma, as animals explored the novel object–place pairings. These results suggest that fast gamma plays a role in encoding memories of novel object–place associations.

## Methods

### Subjects

Ten male Long–Evans rats weighing approximately 350–500 g were used in the study. Rats were housed on a reverse light/dark cycle (lights off from 8:00 A.M. to 8:00 P.M.) and tested during the dark phase. After drive implantation, rats were housed individually in cages (40 × 40 × 40 cm) constructed from clear acrylic and containing enrichment materials (eg, plastic balls, cardboard tubes, and wooden blocks). Rats recovered from surgery for at least 1 week prior to the start of behavioral testing. All experiments were conducted according to the guidelines of the United States National Institutes of Health *Guide for the Care and Use of Laboratory Animals* under an IACUC-approved protocol, in accordance with the Society for Neuroscience’s Policies on the Use of Animals in Neuroscience Research.

### Tetrode and recording drive preparation

Recording drives contained 14 (“hyperdrives”, Gothard et al., 1996; 8 rats) or 26 (Harlan drives, Neuralynx; 2 rats) independently movable tetrodes. Tetrodes were constructed from 17 μm polyimide-coated platinum-iridium (90–10%) wire (California Fine Wire). Electrode tips in tetrodes targeted toward cell body layers were plated with platinum to reduce single channel impedances to ∼150–300 kΩ at 1 kHz.

### Surgery and tetrode placement

Recording drives were surgically implanted above the right hippocampus on the day of surgery. Stereotaxic coordinates were as follows (in mm): 3.8 AP, 3.0 ML, 1.0 DV in nine rats and 5.0 AP, 5.0 ML, 1.0 DV in one rat. In the latter rat, only those tetrodes that were histologically verified to be in dorsal hippocampus were used (ie, the most anterior tetrodes). Bone screws were placed in the skull, and the screws and the base of the drive were covered with dental cement to affix the drive to the skull. Two screws in the skull were connected to the recording drive ground.

Over the course of a few weeks after drive implantation, tetrodes were slowly lowered toward their target locations. In six of the rats implanted with hyperdrives, six tetrodes were targeted toward the CA1 cell body layer and six toward the CA3 cell body layer. In the other four rats, all of the 12 or 24 recording tetrodes were targeted toward the CA1 cell body layer. In each rat, one tetrode was targeted toward the apical dendritic layers of CA1. Another tetrode was used as a reference for differential recording and was placed at the level of the corpus callosum or higher; the reference tetrode was recorded against ground to make sure that it was placed in a quiet location. All recording locations were verified histologically after experiments were finished (see Histology). Representative examples of final recording locations are shown in Figure 1*A*.

### Data acquisition

Data were collected using the Neuralynx data acquisition system. The headstage output of recording drives was conducted via lightweight tether cables through a multichannel slip-ring commutator to a data acquisition system that processed the signals through individual 24 bit AD converters (Digital Lynx, Neuralynx). Unit activity was bandpass filtered from 600 to 6000 Hz, and spike waveforms were time-stamped and recorded at 32 kHz for 1 ms. Local field potentials (LFPs) were recorded continuously in the 0.1–500 Hz band at a sampling rate of 2000 Hz. Notch filters were not used. Continuously sampled LFPs were recorded differentially against a common reference electrode placed in an electrically quiet region (see Surgery and tetrode placement). Light-emitting diodes on the headstages were used to track rats’ movements at a 30 Hz sampling rate.

### Novel object–place association task

On each day of the experiment, animals were allowed to freely explore an open-field environment (60 × 60 cm box) for three 10 min behavioral sessions, alternated with 10 min rest sessions in a towel-lined flower pot (Fig. 1*B*). The open-field environment contained a single index card on the upper edge of one wall to provide a visual orientation cue. Prior to testing, the animal was habituated to the open field for at least 3 d with no objects present. On day 1 of the experiment, two identical objects were placed into the environment in constant locations during all three familiar exploration sessions (“familiarization sessions”). On day 2, the same two object–place pairings were presented during Sessions 1 and 3, but during Session 2, one of the familiar object–place pairings was replaced with a novel object–place pairing. Days 1 and 2 were repeated two additional times to include all three novelty conditions: ie, novel object in constant location (NO), familiar object placed in a location where no object was presented previously (NL), and novel object placed in a location where no object was presented previously (NO+NL). The order of days testing each novelty type, specific location of the objects, and identity of objects were randomly assigned. Objects were built from plastic toy blocks (Legos) and were cleaned after each 10 min exploration session to remove scent cues. Eight rats were tested across all three novelty conditions across successive days, with intervening “re-familiarization” days during which rats again explored the two familiar object–place associations during all three behavioral sessions. Two rats were tested only in the NO+NL and NO conditions and did not have a re-familiarization day between the 2 experimental days.

### Behavioral analysis

The total time during which a rat’s head was within 15 cm of the center of each object during the first 3 min of the 10 min novelty session was determined and used to calculate the discrimination index (DI) between novel and familiar object–place associations [ie, (novel time)/(novel time + familiar time); Fig. 1*C*)]. DI values of ∼0.5 would indicate no preference for the novel object–place association. DI values were also calculated between the two familiar objects in the F condition. DI values from the object exploration conditions were compared with DI values from corresponding locations during sessions in which no objects were present in order to control for innate location preferences.

### Detection of object exploration periods

**Figure 2.**
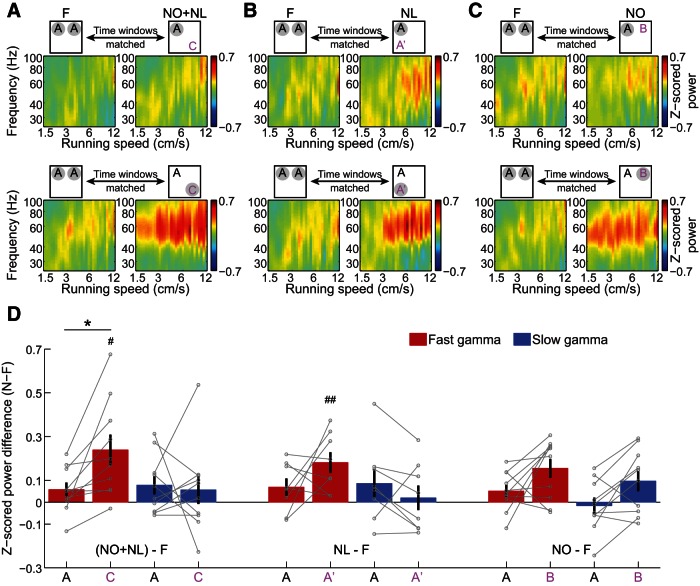
Changes in slow and fast gamma power in CA1 in response to exploration of novel object–place pairings. ***A–C***, Color-coded power across gamma frequencies in CA1 as a function of running speed, plotted during time periods of familiarity exploration versus novelty exploration, averaged across all CA1 tetrodes and rats. The time periods of exploration of familiar object–place pairing A in F conditions were time-matched with those during exploration of familiar object–place pairing A (top row) and novel object–place pairings C, A′, and B (bottom row) in NO+NL (***A***), NL (***B***), and NO (***C***) conditions, respectively. Note that *x*- and *y*-axes are shown in log scale. ***D*,** Changes in fast and slow gamma power between time-matched periods in the F condition and the three novelty conditions (NO+NL, NL, and NO), during exploration of familiar (A) and novel (ie, C, A′, and B) object–place pairings. Data from individual rats are shown in gray. *Indicates significantly (*p* < 0.05) different changes in gamma power from familiarization session to novelty session for exploration of novel object–place pairings compared to exploration of familiar object–place pairings; # and ## indicate that the change in gamma power between N and F sessions was significantly (#*p* < 0.05, ## *p* < 0.01) greater than zero.

**Figure 3.**
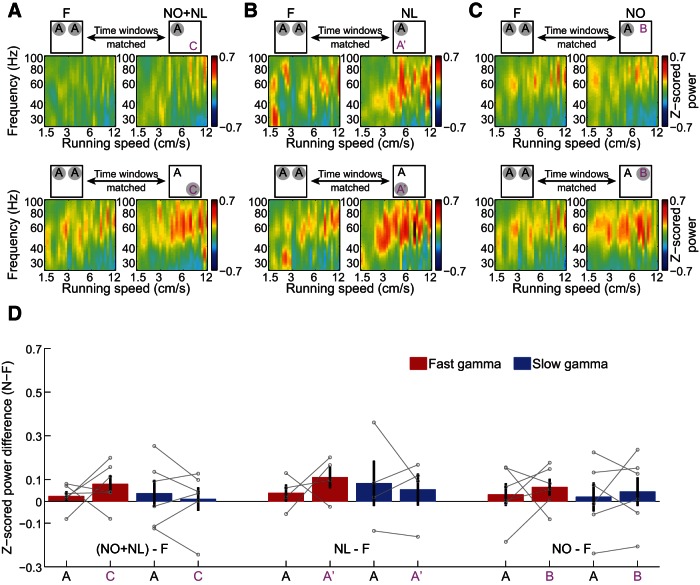
No significant changes in slow and fast gamma power in CA3 during exploration of novel object–place pairings. ***A***–***C***, Same as in Figure 2*A*-*C*, except for CA3 recordings instead of CA1. ***D*,** Changes in fast and slow gamma power in CA3 between time-matched periods in the F condition and the three novelty conditions (NO+NL, NL, and NO) during exploration of familiar (A) and novel (ie, C, A′, and B) object–place pairings. Data from individual rats are shown in gray.

**Figure 4.**
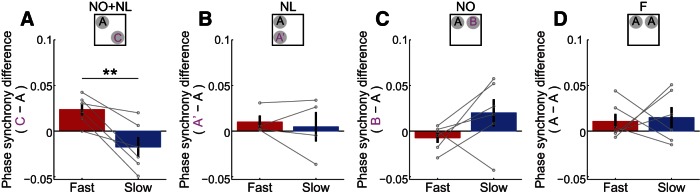
Changes in slow and fast gamma phase synchrony between CA3 and CA1 during exploration of novel object–place pairings. The difference in CA3–CA1 slow and fast gamma phase synchrony between exploration periods for novel and familiar object–place pairings in NO+NL (***A***), NL (***B***), and NO (***C***) conditions. The differences in slow and fast gamma interregional phase synchrony between the explorations periods for the two familiar object–place pairings in the F condition are also shown (***D***). Data from individual rats are shown in gray. **p < 0.01.

**Figure 5.**
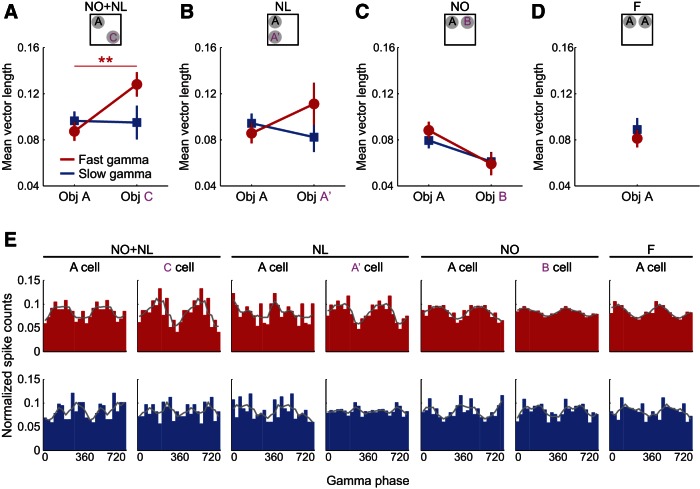
Phase-locking of CA3 and CA1 place cell spikes to CA1 slow and fast gamma during exploration of novel object–place pairings. ***A***–***C***, Mean vector lengths of CA1 slow and fast gamma phase distributions were estimated for spike times of CA3 and CA1 place cells with place fields close to either familiar or novel object–place pairings in the novelty conditions. For the NO+NL condition, place cell spikes were significantly more phase-locked to fast gamma during exploration of the novel object–place pairing than during exploration of the familiar object–place pairing. ***D*,** Mean vector lengths of CA1 slow and fast gamma phase distributions were estimated for spike times of CA3 and CA1 place cells with fields near either of the familiar object–place pairings in the familiar condition. ***E***, Example spike time-gamma phase distributions from individual place cells. Spike counts were normalized (ie, number of spikes in each bin/total spike count). A representative place cell from each cell category is shown for fast gamma (top row, red) and slow gamma (bottom row, blue). Grey lines indicate moving average (moving size = 2 bins). **p < 0.01.

For LFP recording analyses and place cell phase-locking analyses, measures were computed only within time windows when animals were actively exploring an object (see Figs. 2–5, 7). Active object exploration periods for each object in each condition were defined as discrete time windows when a rat’s head was within a 15-cm-diameter circular area around the center of an object, and adjacent time windows were merged if they were separated by <0.5 s. Only the data within the first 30 s time windows of object exploration were used for further analyses to ensure that identical amounts of time were compared across conditions. In addition, to measure how gamma and theta power changed during exploration of novel object–place pairings compared to familiar object–place pairings (see Figs. 2, 3, 7), exploration time windows for the familiar object (Fig. 1*B*, Object A) in the familiar condition (Fig. 1*B*, F) were time-matched to the detected object exploration time windows in the novelty conditions. This was done to ensure that gamma power changes were unaffected by the effects of time within a testing session on gamma power that were shown in a previous study (Bieri et al., 2014). The time matching was performed as follows. In each novel session (ie, Session 2 in NO+NL, NL, and NO conditions), time periods of object–place pairing exploration were identified from the first 30 s of novel object–place pairing exploration and the first 30 s of familiar object–place pairing exploration. For each object–place pairing, the median time point of these discontinuous exploration time windows was obtained. In Session 2 from familiarization and re-familiarization days, time periods of familiar object–place pairing exploration were also identified, thereby producing another series of discontinuous exploration time windows for familiarization and re-familiarization conditions (F). In these familiarization and re-familiarization time windows, the time point that most closely matched the median time point of object–place pairing exploration from the corresponding novelty session was identified and defined as the median time point for each F condition. Time-matched periods of exploration of familiar object–place pairings (ie, either Object A) from each F condition were then defined as the 15 s preceding and following the median time point of object–place pairing exploration in F conditions. This yielded 30-s-long periods of familiar object–place pairing exploration that were time-matched to the 30-s-long periods of object–place pairing exploration in novelty conditions.

A stricter criterion for definition of object exploration was also used for a subset of analyses (see Results and Table 1). This criterion differed from the main criterion for detecting object exploration periods in two ways: (1) a rat’s head was required to be within a 10-cm-diameter circular area around the center of an object, and (2) data within the first 15 s of object exploration were used.

**Table 1:** Statistical table

	Fig.	Description	Data structure	Test	Factor	Degrees of freedom	Statistics value	*p* value
**a**	1C	Discrimination index	Normal distribution	Generalized linear mixed models	Novelty condition × data type interaction	1,72	*F* = 4.568	0.036
					Novelty condition	1, 72	*F* = 5.289	0.024
					Data type	1, 72	*F* = 9.430	0.003
		Discrimination index in Session 2 of all conditions	Normal distribution	Repeated measures ANOVA	Novelty condition	3, 21	*F* = 4.175	0.018 *Post hoc*: NO+NL vs F, *p* = 0.020; NL vs F, *p* = 0.108; NO vs F, *p* = 0.299; NO+NL vs NL, *p* = 0.223; NO+NL vs NO, *p* = 0.037
		Discrimination index in control sessions	Normal distribution	Repeated-measures ANOVA	Novelty condition	3, 21	*F* = 1.809	0.176
		Discrimination index in NO+NL condition	Normal distribution	Paired *t* test	Data type	9	*t* = 4.751	0.001
		Discrimination index in NL condition	Normal distribution	Paired *t* test	Data type	7	*t* = 1.345	0.221
		Discrimination index in NO condition	Normal distribution	Paired *t* test	Data type	9	*t* = 1.259	0.240
		Discrimination index in F condition	Normal distribution	Paired *t* test	Data type	9	*t* = 0.393	0.703
**b**	1D	Exploration time in Session 1	Normal distribution	Repeated-measures ANOVA	Novelty condition	3,21	*F* = 1.216	0.329
**c**	2D,^3^D	Hippocampal gamma power change over running speed in novel and familiar conditions	Normal distribution	Generalized linear mixed models	Running speed	1,5450	*F* = 2.609	0.106
					Brain region	1,5450	*F* = 85.640	<0.001
					Object–place pairing type	1,5450	*F* = 99.128	<0.001
					Gamma type	1,5450	*F* = 66.361	<0.001
		Hippocampal gamma power change in novel and familiar conditions	Normal distribution	Generalized linear mixed models	Interaction: brain region × novelty condition × object–place pairing type × gamma type	1,164	*F* = 3.984	0.048
					Object–place pairing type	1,164	*F* = 7.500	0.007
					Gamma type	1,164	*F* = 3.938	0.049
**d**	2D	CA1 gamma power change between novel and familiar conditions	Normal distribution	Generalized linear mixed models	Interaction: novelty condition × object–place pairing type × gamma type	1,104	*F* = 11.953	0.001
		CA1 gamma power change between NO+NL and F conditions	Binomial distribution	Binomial test		N/A	N/A	Fast gamma power change: Object C: *p* = 0.021; Object A: *p* = 0.109; Slow gamma power change: Object C: *p* = 0.754; Object A: *p* = 0.754
		CA1 gamma power change between NO+NL and F conditions	Normal distribution	Generalized linear mixed models	Interaction: object–place pairing type × gamma type	1,36	*F* = 6.941	0.012 *Post hoc*: Fast gamma: Obj A vs C, *p* = 0.011; Slow gamma: Obj A vs C, *p* = 0.791
		CA1 gamma power change between NL and F conditions	Binomial distribution	Binomial test		N/A	N/A	Fast gamma power change: Object A’: *p* =0.008; Object A: *p* =0.289; Slow gamma power change: Object A’: *p* =1.000; Object A: *p* = 0.289
		CA1 gamma power change between NL and F conditions	Normal distribution	Generalized linear mixed models	Interaction: object–place pairing type × gamma type	1,28	*F* = 6.109	0.020 *Post hoc*: Fast gamma: Obj A vs A’, *p* = 0.169; Slow gamma: Obj A vs A’, *p* = 0.226
		CA1 gamma power change between NO and F conditions	Binomial distribution	Binomial test		N/A	N/A	Fast gamma power change: Object B: *p* = 0.109; Object A: *p* = 0.109; Slow gamma power change: Object B: *p* = 0.754; Object A: *p* = 0.754

		CA1 gamma power change between NO and F conditions	Normal distribution	Generalized linear mixed models	Object–place pairing type	1,36	*F* = 1.406	0.243
					Gamma type	1,36	*F* = 3.174	0.083
					Interaction: object–place pairing type × gamma type	1,36	*F* = 0.054	0.817 *Post hoc*: Fast gamma: Obj A vs B, *p* = 0.090; Slow gamma: Obj A vs B, *p* = 0.025
		CA1 gamma power change between novel and familiar conditions, using stricter criterion of exploration	Normal distribution	Generalized linear mixed models	Interaction: novelty condition × object–place pairing type × gamma type	1,104	*F* = 5.087	0.026
		CA1 gamma power change between NO+NL and F conditions, using stricter criterion of exploration	Binomial distribution	Binomial test		N/A	N/A	Fast gamma power change: Object C: *p* = 0.021; Object A: *p* = 0.344; Slow gamma power change: Object C: *p* = 0.344; Object A: *p* = 1.000
		CA1 gamma power change between NO+NL and F conditions, using stricter criterion of exploration	Normal distribution	Generalized linear mixed models	Interaction: object–place pairing type × gamma type	1,36	*F* = 3.953	0.054 *Post hoc*: Fast gamma: Obj A vs C, *p* = 0.016; Slow gamma: Obj A vs C, *p* = 0.354
		CA1 gamma power change between NL and F conditions, using stricter criterion of exploration	Normal distribution	Generalized linear mixed models	Interaction: object–place pairing type × gamma type	1,28	*F* = 4.241	0.049 *Post hoc*: Fast gamma: Obj A vs A’, *p* = 0.060; Slow gamma: Obj A vs A’, *p* = 0.806
		CA1 gamma power change between NO and F conditions, using stricter criterion of exploration	Normal distribution	Generalized linear mixed models	Object–place pairing type	1,36	*F* = 0.527	0.473
					Gamma type	1,36	*F* = 0.068	0.796
					Interaction: object–place pairing type × gamma type	1,36	*F* = 0.107	0.745 *Post hoc*: Fast gamma: Obj A vs B, *p* = 0.129; Slow gamma: Obj A vs B, *p* = 0.224
**e**	3D	CA3 gamma power change between NO+NL and F conditions	Binomial distribution	Binomial test		N/A	N/A	Fast gamma power change: Object C: *p* = 0.219; Object A: *p* = 0. 219; Slow gamma power change: Object C: *p* = 0.219; Object A: *p* = 1.000
		CA3 gamma power change between NL and F conditions	Binomial distribution	Binomial test		N/A	N/A	Fast gamma power change: Object A’: *p* = 0.625; Object A: *p* = 0.625; Slow gamma power change: Object A’: *p* = 0.625; Object A: *p* = 0.625
		CA3 gamma power change between NO and F conditions	Binomial distribution	Binomial test		N/A	N/A	Fast gamma power change: Object B: *p* = 0.219; Object A: *p* = 0.688; Slow gamma power change: Object B: *p* = 0.688; Object A: *p* = 1.000
		CA3 gamma power change between novel and familiar conditions	Normal distribution	Generalized linear mixed models	Interaction: novelty condition × object–place pairing type × gamma type	1,56	*F* = 1.138	0.291
					Interaction: object–place pairing type × gamma type	1,56	*F* = 1.161	0.286
					Novelty condition	1,56	*F* = 0.266	0.608

					Object–place pairing type	1,56	*F* = 0.984	0.325
					Gamma type	1,56	*F* = 0.520	0.474
		CA3 gamma power change between NO+NL and F conditions	Normal distribution	Generalized linear mixed models	Interaction: object–place pairing type × gamma type	1,20	*F* = 1.045	0.319 *Post hoc*: Fast gamma: Obj A vs C, *p* = 0.372; Slow gamma: Obj A vs C, *p* = 0.589
**f**	4	Gamma phase synchrony change between novel and familiar object–place pairings	Normal distribution	Generalized linear mixed models	Interaction: novelty condition × gamma type	1,40	*F* = 11.005	0.002
		Gamma phase synchrony difference (C–A)	Normal distribution	Paired *t* test	Gamma type	5	*t* = 4.316	0.008
		Gamma phase synchrony difference (A’–A)	Normal distribution	Paired *t* test	Gamma type	3	*t* = 0.420	0.703
		Gamma phase synchrony difference (B–A)	Normal distribution	Paired *t* test	Gamma type	5	*t* = 1.707	0.148
**g**	5	Mean vector length of gamma phase distributions	Normal distribution	Generalized linear mixed models	Interaction: novelty condition × cell type × gamma type	1,398	*F* = 3.980	0.047
	5A	Mean vector length of gamma phase distributions in NO+NL condition	Normal distribution	Generalized linear mixed models	Interaction: cell type × gamma type	1,110	*F* = 4.812	0.030 *Post hoc*: Fast gamma: cells A vs cells C, *p* = 0.008; Slow gamma: cells A vs cells C, *p* = 0.928
	5B	Mean vector length of gamma phase distributions in NL condition	Normal distribution	Generalized linear mixed models	Interaction: cell type × gamma type	1,100	*F* = 4.136	0.045 *Post hoc*: Fast gamma: cells A vs cells A’, *p* = 0.159; Slow gamma: cells A vs cells A’, *p* = 0.428
	5C	Mean vector length of gamma phase distributions in NO condition	Normal distribution	Generalized linear mixed models	Interaction: cell type × gamma type	1,132	*F* = 0.416	0.520 *Post hoc*: Fast gamma: cells A vs cells B, *p* = 0.049; Slow gamma: cells A vs cells B, *p* = 0.163
**h**	6D	Place cell in-field firing rates in all novel conditions	Normal distribution	Generalized linear mixed models	Interaction: novelty condition × cell type × gamma type	1,146	*F* = 0.549	0.460
					Interaction: cell type × gamma type	1,146	*F* = 4.538	0.035
		Place cell in-field firing rates in NO+NL condition	Normal distribution	Generalized linear mixed models	Interaction: cell type × gamma type	1,56	*F* = 4.507	0.038 *Post hoc* (sign test): Cell C: slow vs fast gamma, *p* = 0.039; Cell A: slow vs fast gamma, *p* = 0.238
		Place cell in-field firing rates in NL condition	Normal distribution	Generalized linear mixed models	Interaction: cell ype × gamma type	1,38	*F* = 33.532	<0.001 *Post hoc* (sign test): Cell A’: slow vs fast gamma, *p* < 0.008; Cell A: slow vs fast gamma, *p* = 0.092
		Place cell in-field firing rates in NO condition	Normal distribution	Generalized linear mixed models	Interaction: cell type × gamma type	1,48	*F* = 1.322	0.256
					Cell type	1,48	*F* = 0.015	0.902
					Gamma type	1,48	*F* = 0.005	0.942
**i**	6E	Place cell in-field firing rates in NO+NL condition (gamma detected by non-local EEG)	Normal distribution	Sign test	Gamma type	N/A	N/A	Cell C: slow vs fast gamma, *p* = 0.039; Cell A: slow vs fast gamma, *p* = 0.481
		Place cell in-field firing rates in NL condition (gamma detected by non-local EEG)	Normal distribution	Sign test	Gamma type	N/A	N/A	Cell A’: slow vs fast gamma, *p* = 0.070; Cell A: slow vs fast gamma, *p* = 0.267

**j**	7D,E	Hippocampal theta power change between novel and familiar conditions	Normal distribution	Generalized linear mixed models	Interaction: brain region × novelty condition × object–place pairing type	1,80	*F* = 0.001	0.976
	7D	CA1 theta power change between novel and familiar conditions	Normal distribution	Generalized linear mixed models	Interaction: novelty condition × object–place pairing type	1,52	*F* = 3.410	0.070
		CA1 theta power change between NO+NL and F conditions	Normal distribution	Paired *t* test	Object–place pairing type	9	*t* = 1.317	0.220
		CA1 theta power change between NL and F conditions	Normal distribution	Paired *t* test	Object–place pairing type	7	*t* = 1.986	0.087
		CA1 theta power change between NO and F conditions	Normal distribution	Paired *t* test	Object–place pairing type	9	*t* = 0.041	0.968
	7E	CA3 theta power change between novel and familiar conditions	Normal distribution	Generalized linear mixed models	Interaction: novelty condition × object–place pairing type	1,28	*F* = 0.650	0.427
		CA3 theta power change between NO+NL and F conditions	Normal distribution	Paired *t* test	Object–place pairing type	5	*t* = 1.934	0.111
		CA3 theta power change between NL and F conditions	Normal distribution	Paired *t* test	Object–place pairing type	3	*t* = 1.109	0.348
		CA3 theta power change between NO and F conditions	Normal distribution	Paired *t* test	Object–place pairing type	5	*t* = 1.849	0.124
	N/A	Hippocampal theta power change between novel and familiar conditions, using stricter criterion of exploration	Normal distribution	Generalized linear mixed models	Interaction: brain region × novelty condition × object–place pairing type	1,80	*F* = 0.049	0.825
		CA1 theta power change between novel and familiar conditions, using stricter criterion of exploration	Normal distribution	Generalized linear mixed models	Interaction: novelty condition × object–place pairing type	1,52	*F* = 0.013	0.910
		CA1 theta power change between NO+NL and F conditions, using stricter criterion of exploration	Normal distribution	Paired *t* test	Object–place pairing type	9	*t* = 1.287	0.230
		CA1 theta power change between NL and F conditions, using stricter criterion of exploration	Normal distribution	Paired *t* test	Object–place pairing type	7	*t* = 2.040	0.081
		CA1 theta power change between NO and F conditions, using stricter criterion of exploration	Normal distribution	Paired *t* test	Object–place pairing type	9	*t* = 0.793	0.448
**k**	7F	Theta phase synchrony change between novel and familiar object–place pairings	Normal distribution	Repeated-measures ANOVA	Novelty condition	3,9	*F* = 2.484	0.127
**l**	7G	Mean vector length of theta phase distributions	Normal distribution	Two-way ANOVA	Interaction: novelty condition × cell type	2,195	*F* = 3.085	0.048
		Mean vector length of theta phase distributions in NO+NL condition	Normal distribution	*t* test	cell type	55	*t* = 2.192	0.033
		Mean vector length of theta phase distributions in NL condition	Normal distribution	*t* test	cell type	50	*t* = 0.808	0.423
		Mean vector length of theta phase distributions in NO condition	Normal distribution	*t* test	cell type	66	*t* = 0.966	0.338

### Estimation of running speed

The running speed ($v_{t}$) at time point (t) was estimated by calculating the distance between the preceding position ($x_{t-1},{\;y}_{t-1}$) and the following position ($x_{t+1},{\;y}_{t+1}$), and dividing by the elapsed time (2 × 1/position sampling frequency). The sampling frequency of the position data was 30 Hz, yielding a temporal resolution of 1/15 s (see Figs. 2, 3, 7).

### Estimation of power spectra across running speeds during object exploration

The power spectra were measured across different running speeds as described previously (Ahmed and Mehta, 2012; Zheng et al., 2015; see Figs. 2, 3, 7). Briefly, the absolute power spectrum was calculated for successive 200 ms time windows of the LFP recordings in 10 min sessions, using the multitaper spectral analysis (Mitra and Bokil, 2008) in the Chronux toolbox (http://chronux.org/). Then, the absolute power for each frequency was Z-scored across time for the LFP recording from each tetrode, in order to allow for comparisons across different frequencies that would otherwise be difficult due to the 1/f decay of power in physiological signals. Running speed was calculated (see Estimation of running speed) and averaged within each 200 ms time window corresponding to the LFP segments. To produce power estimates across running speed bins, Z-scored absolute power at each frequency was averaged across all time windows that fell within a given speed bin and smoothed with a Gaussian kernel centered on that bin. Speed and frequency were plotted on a log-log scale for gamma frequencies (Figs. 2, 3), which allows for better visualization of the relatively narrow band of slow gamma frequencies (ie, compared to the fast gamma band) and the reduced range of running speeds associated with slow gamma compared to fast gamma (Ahmed and Mehta, 2012; Zheng et al., 2015).

### CA3–CA1 phase synchrony

Time-varying phase synchrony between areas CA1 and CA3 was calculated using a previously introduced method (Lachaux et al., 1999; see Figs. 4, 7*F*). This method assesses covariance between the instantaneous phases of each oscillation frequency for a pair of recordings by measuring the variability of phase differences between the recordings. Phase was calculated for each frequency of interest as a function of time by computing the convolution of the signal with complex Morlet’s wavelets. The phase of this convolution $\varphi \left(t\right)$ was then extracted for all time points t for each recording. Phase synchrony (PS) within each object exploration time window was then determined for each frequency by taking the average value at each time point:

$$PS=\;\frac{1}{n}\left|\sum_{t=t_{1}}^{t_{n}}exp\left(j\theta \left(t\right)\right)\right|,$$

where $\theta \left(t\right)$ is the phase difference between the two signals $\varphi_{1}\left(t\right)-\varphi_{2}\left(t\right)$ (ie, the phase difference between CA3 and CA1 signals) at each time point. If phase differences between recording pairs remain relatively constant across time, then the two signals are defined as phase synchronous and PS values would be close to 1.

Phase synchrony measures for slow gamma, fast gamma, and theta were estimated during object exploration time periods (defined in Detection of object exploration periods). For each object exploration window, a single theta, slow gamma, and fast gamma phase synchrony measure was found by averaging phase synchrony estimates across time, across frequencies within each respective frequency range (ie, 6–12 Hz for theta, 25–55 Hz for slow gamma, and 60–100 Hz for fast gamma), and lastly across all CA1 and CA3 recording pairs associated with the object exploration window.

### Place cell phase-locking

The main place field of a place cell was identified as the collection of contiguous spatial bins (3 × 3 cm) in which the firing rate was greater than 0.4 × the peak firing rate across the whole session. For each place field, a peak firing position was determined (ie, the position within the field that exhibited the maximum firing rate). A total of 131 place cells (*n* = 98 CA1 cells and 33 CA3 cells) with a place field peak firing position located less than 20 cm away from the center of either object were identified and included in this study. Only the spike times occurring in locations within 15 cm of the center of either object during object exploration time windows (described in Detection of object exploration periods) were included in this analysis. The time-varying phases for theta, slow gamma, and fast gamma were determined using the Hilbert transform of the bandpass filtered signal for each respective frequency range. The theta, slow gamma, and fast gamma spike phase distributions for each cell were then determined by identifying the theta, slow gamma, and fast gamma phases, respectively, at the EEG time point closest to each spike time. Phase-locking was quantified using the mean vector length of the resulting phase distributions (see Figs. 5, 7G). For all CA1 and CA3 place cells, phases for each spike time were estimated for CA1 LFPs from all simultaneously recorded CA1 tetrodes that picked up single units.

### Reconstruction of place fields during slow and fast gamma

**Figure 6.**
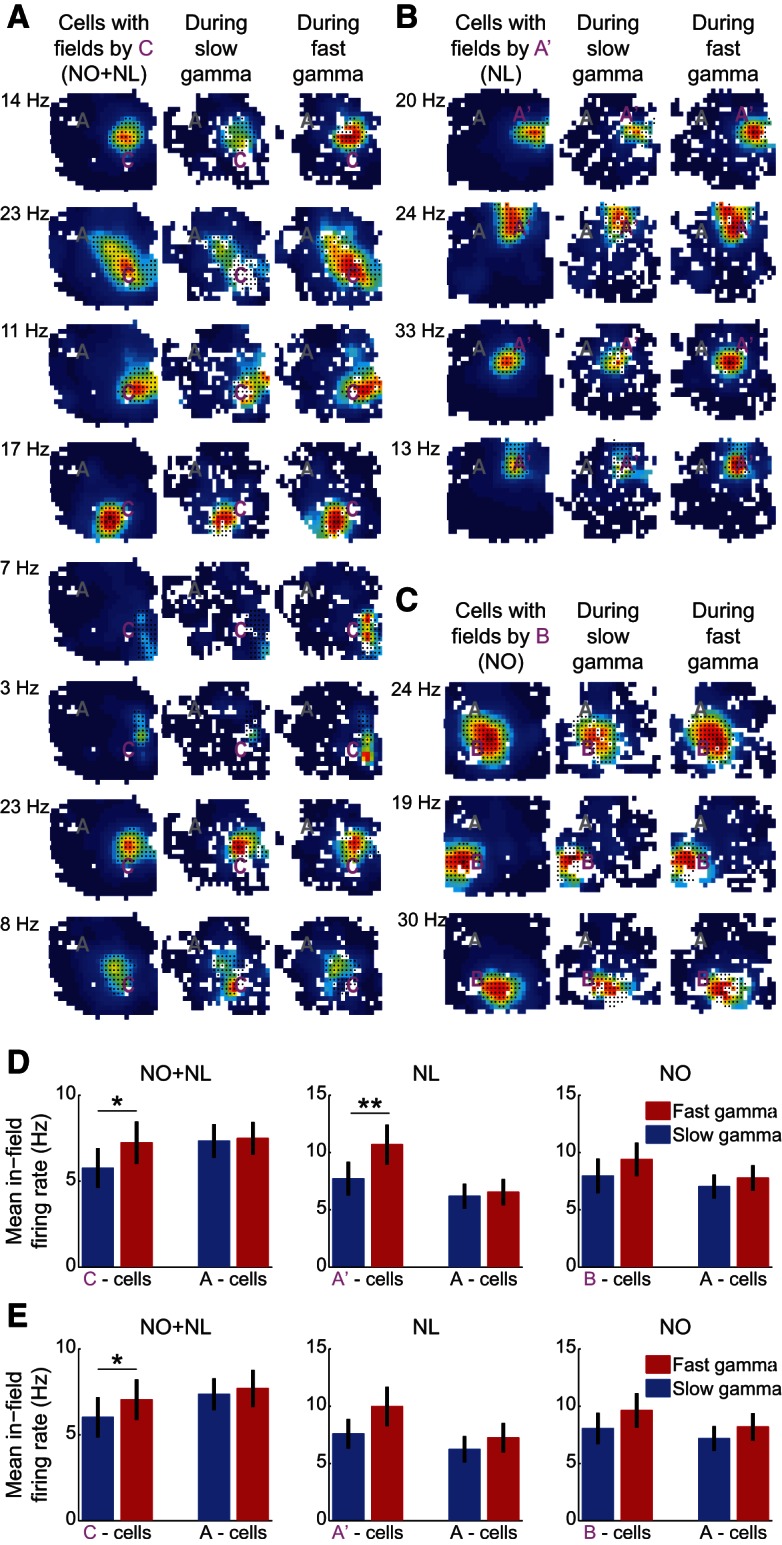
CA1 place cell spiking increased selectively in fast gamma periods during exploration of novel object–place pairings. ***A***–***C***, Examples of color-coded rate maps of CA1 place cells that exhibited place fields close to the novel object–place pairs in the NO+NL (***A***), NL (***B***), and NO (***C***) conditions. Red indicates peak firing rate, dark blue represents no firing, and white pixels indicate unvisited areas. Rate maps constructed from spikes across the entire exploration session are shown in the left columns. Rate maps constructed from spike times during slow and fast gamma episodes are shown in the middle and right columns, respectively. Black dots indicate the defined place fields. Each map is shown scaled to the peak firing rate of the cell across the entire session, which is shown to the left. ***D***, Mean in-field firing rates of CA1 place cells during slow and fast gamma episodes that occurred during exploration of novel or familiar object–place pairings. In these plots, slow and fast gamma episodes were detected from the same tetrodes on which the cells were recorded. ***E***, The same as ***D***, except that slow and fast gamma were detected using different tetrodes than the ones on which cells were recorded. *p < 0.05, **p < 0.01.

In each 10 min recording session, all successive 200 ms time windows were ranked according to their peak slow gamma power and peak fast gamma power (ranked separately for slow and fast gamma. A rank of 0 corresponded to lowest power, and a rank of 1 corresponded to highest power. Slow gamma windows and fast gamma windows were then defined as those time windows exhibiting power rank values for the gamma type of interest that were >0.5 and power rank values for the other gamma type that were <0.5. For each CA1 place cell, the rate map was then reconstructed by using the spike times occurring only during slow gamma windows or only during fast gamma windows (see Fig. 6). Out of all of the place cells identified as described in the “Place cell phase-locking” section, only those place cells exhibiting relatively intact firing maps during both slow and fast gamma were included. Relatively intact firing maps were defined as those maps in which the intersection area of the place field between reconstructed and raw firing maps was >20% of the area of the raw place field.

### Statistics

**Figure 7.**
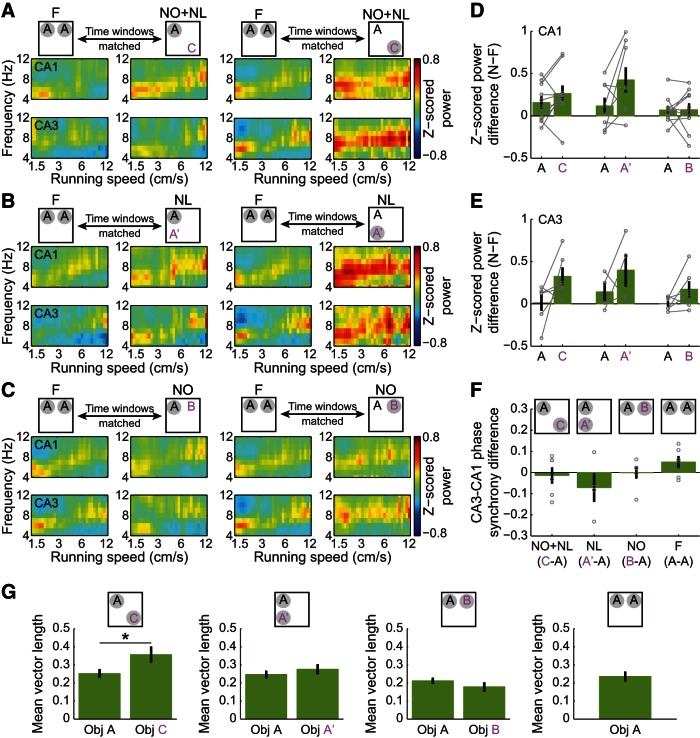
Changes in theta power, CA3–CA1 phase synchrony, and place cell firing patterns during exploration of novel object–place pairings. ***A***–***C***, Color-coded theta power in CA1 (top rows) and CA3 (bottom rows) as a function of running speed during exploration of familiar and novel object–place pairings, averaged across all recordings for each region. As in Figures 2 and 3, the familiar object–place pair exploration periods in the F condition (first and third columns) were time-matched with those during exploration of familiar object–place pairs (second column) and novel object–place pairs (fourth column) in NO+NL (***A***), NL (***B***), and NO (***C***) conditions. ***D***, ***E***, No significant changes in CA1 (***D***) and CA3 (***E***) theta power occurred between time-matched periods in the F condition and the three novelty conditions (NO+NL, NL, and NO) during exploration of familiar and novel object–place pairings. Data from individual rats are shown in gray. ***F***, CA3–CA1 theta phase synchrony did not significantly change between novel and familiar object–place pair exploration in NO+NL, NL, and NO conditions, nor between explorations of the two familiar object–place pairs in the F condition. Data from individual rats are shown in gray. ***G***, Mean vector lengths of CA1 theta phase distributions for CA3 and CA1 place cell spike times in novelty and familiarization conditions. Theta phase-locking was higher during novel object exploration compared to familiar object exploration for the NO+NL condition. *p < 0.05.

Statistics were computed using SPSS 22 (IBM). Generalized linear mixed models were used to test for effects of novelty condition (NO+NL, NL, NO, or F) and data type (objects vs no objects) on the discrimination index behavioral measure (Fig. 1*C*), with repeated-measures ANOVAs used as *post hoc* tests. A repeated-measures ANOVA was also used to test for differences in the average duration of active object exploration across novelty conditions (Fig. 1*D*). Generalized linear mixed models were also used to assess effects of brain area (CA1 or CA3), novelty condition (NO+NL, NL, NO, or F), object–place pairing type (novel or familiar), gamma type (slow or fast gamma), and place cell type (cells with place fields close to novel or familiar objects) on physiology measures (see Figs. 2–6, 7). Paired *t* tests were used as *post hoc* tests for gamma power (Figs. 2, 3) and phase synchrony (Fig. 4) measures. Binomial tests were performed to assess whether gamma power increases during exploration of novel object–place pairings were significantly greater than zero (Figs. 2,3). *t* Tests were used as *post hoc* tests for gamma phase-locking of place cell spikes (Fig. 5). Sign tests were used as *post hoc* tests to assess whether place cells with fields near novel objects exhibited higher firing rates during fast gamma periods than during slow gamma periods (Fig. 6). Paired *t* tests were used as post hoc tests for theta power (Fig. 7*D,E*). *t* Tests were used as post hoc tests for theta phase locking of place cell spikes (Fig. 7*G*). For theta phase synchrony (Fig. 7*F*), a repeated-measures ANOVA was performed. Data are shown as mean ± SEM, unless indicated otherwise.

### Histology

For verification of tetrode locations, brains were cut coronally into 30 μm sections and stained with cresyl violet (Fig. 1*A*). All tetrode tracks were identified, and the deepest location of each tetrode was determined by comparison across adjacent sections.

## Results

Continuously sampled LFP recordings and place cell spike trains were obtained from strata pyramidale of hippocampal subfields CA1 and CA3 of six rats and CA1 of an additional four rats (Fig. 1*A*) performing a novel object–place association task (Fig. 1*B*). Three types of novelty were examined: a change in NO, a change in NL, and a change in NO+NL. Behavioral effects of novelty were determined using a DI that compared exploration of novel and familiar object–place pairings [novel time/(novel time + familiar time)] in sessions containing objects; control DI values were defined from corresponding locations during sessions on earlier days in which no objects were present in the testing arena. The behavioral effect of novelty differed across conditions and was not explained by innate location preferences, as evidenced by a significant interaction between, and significant main effects of, novelty condition and data type (ie, experimental “Objects” sessions or control “No objects” sessions) on the DI (Fig. 1*C*; interaction, *F*_(1,72)_ = 4.6, *p* = 0.04^a^; main effect of novelty condition, *F*_(1,72)_ = 5.3, *p* = 0.03^a^; main effect of data type, *F*_(1,72)_ = 9.4, *p* = 0.003^a^, generalized linear mixed models, *n* = 10 rats in F, NO+NL, and NO conditions, and *n* = 8 rats in NL condition; superscript letters following *p* values correspond to statistics presented in Table 1). In experimental Objects sessions, there was a significant effect of novelty condition on DI values (*F*_(3,21)_ = 4.2, *p* = 0.02^a^, repeated-measures ANOVA); however, no effect of novelty condition was found on DI values in control No objects sessions (*F*_(3,21)_ = 1.8, *p* = 0.2^a^, repeated-measures ANOVA). Periods of exploration of novel object–place pairings in Session 2 of NO+NL, NL, and NO were compared to exploration of familiar object–place pairings in Session 2 of familiarization and re-familiarization days (F). Rats explored novel objects in new locations more than they explored familiar objects during familiarization and re-familiarization days (NO+NL vs F, *p* = 0.02^a^, *post hoc* tests for repeated-measures ANOVA). Rats also explored novel objects in new locations more than they explored novel objects in familiar locations (NO+NL vs NO, *p* = 0.04^a^, *post hoc* tests for repeated-measures ANOVA) and more than they explored the same locations in sessions in which no objects were presented (Objects vs No objects, *t*_(9)_ = 4.8, *p* = 0.001^a^, paired *t* test). Significant novelty effects on behavior were not obtained for the NL condition or NO condition, however (NL vs F, *p* = 0.1, NO vs F, *p* = 0.3^a^, *post hoc* test for repeated-measures ANOVA). The lack of significant novelty effects on behavior for the NL and NO conditions was not explained by lower levels of familiar exploration during Session 1 in the NL and NO conditions compared to the NO+NL condition (Fig. 1*D*; *F*_(3,21)_ = 1.2, *p* = 0.3^b^; repeated-measures ANOVA). It is thus possible that the NO and NL conditions were insufficiently novel for piquing rats’ curiosity, especially in the NO condition given that novel and familiar objects were constructed from the same materials (ie, Lego toy blocks).

Next, slow and fast gamma rhythms in CA1 and CA3 were compared during exploration of novel and familiar object–place pairings (Figs. 2, 3). Slow and fast gamma power estimates were plotted against running speed to examine whether effects were related to differences in running speed, considering that slow and fast gamma are differentially affected by running speed (Ahmed and Mehta, 2012; Kemere et al., 2013; Zheng et al., 2015). Time windows within novel and familiar sessions were also time-matched to account for changes in gamma power that occur across time within a testing session (Bieri et al., 2014; see Materials and Methods, Detection of object exploration periods). For the results described below, only data from Session 2 were analyzed because novel object–place pairing exploration always occurred in Session 2 (Fig. 1*B*). Time windows were selected to be 30 s in duration because ∼30 s of object–place pairing exploration occurred during the first 3 min in Session 2 of each condition (Fig. 1*E*), and these 3 min periods were used to assess behavioral effects of novelty (see Materials and Methods, Behavioral analysis). The change in gamma power during exploration of novel object–place pairings in novel sessions compared with time-matched periods of exploration of familiar object–place pairings in familiar sessions was then measured. This gamma power difference was measured for each hippocampal subregion (ie, CA3 vs CA1), each novelty condition (ie, NO+NL, NL, or NO), each object–place pairing type (ie, novel vs familiar), and each gamma type (ie, slow vs fast). There was no effect of running speed on the gamma power difference between novel and familiar sessions (*F*_(1,5450)_ = 2.6, *p* = 0.1^c^, generalized linear mixed models), and thus measures were averaged across running speeds in subsequent analyses. A significant interaction of hippocampal subregion, novelty condition, object–place pairing type, and gamma type (*F*_(1,164)_ = 4.0, *p* = 0.05^c^, generalized linear mixed models, *n* = 10 rats in F, NO+NL, and NO conditions and *n* = 8 rats in the NL condition), and significant main effects of object–place pairing type (*F*_(1,164)_ = 7.5, *p* = 0.007^c^) and gamma type (*F*_(1,164)_ = 4.0, *p* = 0.05^c^), were obtained. Recordings from CA1 (Fig. 2) and CA3 (Fig. 3) were then analyzed separately, as described below.

Whether CA1 gamma power during object exploration changed between novel and familiar sessions depended on which novelty condition was assessed, whether the object–place pairing was familiar or novel, and which type of gamma was measured (interaction: *F*_(1,104)_ = 12.0, *p* = 0.001^d^, generalized linear mixed models, *n* = 10 rats in F, NO+NL, and NO conditions, and *n* = 8 rats in NL condition). For the NO+NL condition, CA1 power in the fast but not slow gamma range increased across a broad range of running speeds when animals explored novel objects in novel locations (Fig. 2*A*). The difference between fast gamma power during novel object exploration in the NO+NL session and fast gamma power during familiar object exploration in F sessions was significantly >0 (Fig. 2*D*; Object C, *p* = 0.02^d^, Binomial test on *n* = 10 rats). This effect was not observed for exploration of the familiar object in the NO+NL session (Fig. 2*D*; Object A, *p* = 0.1^d^; Binomial test on *n* = 10 rats). This indicates that fast gamma power increased selectively during exploration of the novel object in the NO+NL session. Corresponding effects were not observed for slow gamma (Fig. 2*D*; Object C, *p* = 0.8^d^, Object A, *p* = 0.8; Binomial test on *n* = 10 rats). Accordingly, there was a significant interaction between gamma type and object type (ie, novel Object C or familiar Object A) on gamma power increases during the NO+NL session relative to F sessions (Fig. 2*D*; *F*_(1,36)_ = 7.0, *p* = 0.01^d^, generalized linear mixed models), indicating that slow and fast gamma power changed differently during exploration of novel object–place pairings. Relative to fast gamma power in F sessions, fast gamma power during novel object exploration in NO+NL increased significantly more than fast gamma power during familiar object exploration in NO+NL (Fig. 2*D*; *p* = 0.01^d^, *post hoc* for generalized linear mixed models). Analogous results were not observed for slow gamma (Fig. 2*D*; *p* = 0.8^d^, *post hoc* for generalized linear mixed models). These results indicate that fast, but not slow, gamma was enhanced during novel, but not familiar, object exploration in the NO+NL session. The same pattern of results was observed when a stricter criterion was used to define object exploration (see Materials and Methods; Table 1 ^d^).

Fast, but not slow, gamma power in CA1 increased during exploration of the novel object–place pairing (A′) in NL relative to fast gamma power during familiar object exploration in F (Fig. 2*B*,*D*; fast gamma, *p* = 0.008^d^; slow gamma, *p* = 1.0^d^; Binomial test on *n* = 8 rats). However, gamma power changes during exploration of the novel object–place pairing in NL were not significantly different than gamma power changes during exploration of the familiar object–place pairing in NL (Fig. 2B,*D*; interaction between object–place pairing type and gamma type: *F*_(1,28)_ = 6.1, *p* = 0.02^d^; fast gamma, *p* = 0.2^d^; slow gamma, *p* = 0.2^d^; generalized linear mixed models, *n* = 8 rats). Moreover, significant behavioral effects were not observed in the NL condition (ie, rats did not appear to robustly discriminate between novel and familiar object–place pairings in NL; Fig. 1*C*). For this reason, it is unclear whether or not rats recognized the novel object–place pairing in NL as novel, making interpretation of the gamma results for the NL condition problematic.

For the NO condition, neither fast nor slow gamma power increased significantly during novel object exploration in the NO session relative to exploration of familiar objects in F sessions (Fig. 2*D*; *p* = 0.1^d^ for fast gamma and *p* = 0.8^d^ for slow gamma; Binomial tests on *n* = 10 rats). Also, there was no significant object type × gamma type interaction and no significant main effects on power changes during novel object exploration compared to familiar object exploration in the NO session (Fig. 2C,*D*; interaction between object type and gamma type: *F*_(1,36)_ = 0.05, *p* = 0.8^d^; main effect of object type: *F*_(1,36)_ = 1.4, *p* = 0.2^d^; main effect of gamma type: *F*_(1,36)_ = 3.2, *p* = 0.08^d^; generalized linear mixed models, *n* = 10 rats). It should be noted that significant behavioral effects were not observed in the NO condition (ie, rats did not appear to discriminate between novel and familiar objects when locations remained constant; Fig. 1*C*), and thus it is possible that animals did not recognize the changed object as novel. For this reason, the lack of gamma effects in the NO condition are difficult to interpret.

CA3 has been proposed to be critical for associative memory (McNaughton and Morris, 1987; Treves and Rolls, 1994; Hasselmo et al., 1995; Levy, 1996). Thus, encoding or retrieval of associations between objects and locations may involve CA3. However, no significant slow nor fast gamma power changes were found in CA3 in any of the novel conditions during exploration of novel object–place pairings relative to exploration of familiar object–place pairings in familiar conditions (Fig. 3; NO+NL: slow gamma, *p* = 0.2^e^, fast gamma, *p* = 0.2^e^, *n* = 6; NL: slow gamma, *p* = 0.6^e^; fast gamma, *p* = 0.6^e^, n = 4; NO: slow gamma, *p* = 0.7^e^; fast gamma, *p* = 0.2^e^, *n* = 6; Binomial test). Accordingly, CA3 gamma power during object exploration was not found to significantly change between novel and familiar conditions, regardless of novelty condition, object–place pairing type, and gamma type (novelty condition × object–place pairing type × gamma type interaction: *F*_(1,56)_ = 1.1, *p* = 0.3^e^; novelty condition: *F*_(1,56)_ = 0.3, *p* = 0.6^e^; object–place pairing type, *F*_(1,56)_ = 1.0, *p* = 0.3^e^; gamma type: *F*_(1,56)_ = 0.5, *p* = 0.5^e^; generalized linear mixed models). Also, unlike for CA1, CA3 fast gamma power in the NO+NL session did not increase more, relative to CA3 fast gamma power in F sessions, during exploration of novel object–place pairings compared to familiar object place pairings (NO+NL: fast gamma, *p* = 0.4^e^, *post hoc* for mixed models, *n* = 6 rats). However, it is possible that CA3 effects were not detected due to the lower number of CA3 recordings compared with CA1 recordings (ie, CA3 recordings from 6 rats and CA1 recordings from 10 rats).

It may also be possible, though, that analogous effects in CA3 were not detected because of the nature of LFP signals in CA3. The curve of the cell body layer in CA3 may cause currents to flow in different directions. This may prevent currents from summing together nicely to generate a large and easily detectable LFP. Thus, slow and fast gamma coupling between CA1 and CA3 was also examined by estimating phase synchrony, which measures the consistency of phase differences between two signals and thus is potentially less affected by low amplitude LFPs (Fig. 4). CA3–CA1 phase synchrony results were consistent with CA1 power effects reported above. Specifically, there was a significant interaction between gamma type and novelty condition on the change in gamma phase synchrony during explorations of novel object–place pairings compared to familiar object–place pairings, indicating that CA3–CA1 slow and fast gamma oscillatory coupling were differentially affected by novelty conditions (Fig. 4; *F*_(1,40)_ = 11.0, *p* = 0.002^f^, generalized linear mixed models, *n* = 6 rats in F, NO+NL, and NO conditions, and *n* = 4 rats in NL condition). In the NO+NL session, the increase in fast gamma coupling during novel object exploration relative to familiar object exploration was significantly greater than the corresponding change in slow gamma coupling (Fig. 4*A*; *t*_(5)_ = 4.3, *p* = 0.008^f^, paired *t* test, *n* = 6 rats). In the NL and NO sessions, no significant differences were observed between slow and fast gamma for phase synchrony measures (Fig. 4*B,C*; NL: *t*_(3)_ = 0.4, *p* = 0.7^f^, *n* = 4 rats; NO: *t*_(5)_ = 1.7, *p* = 0.1^f^, *n* = 6 rats; paired *t* test). These results raise the possibility that enhanced fast gamma coupling between CA3 and CA1 facilitates encoding of memories of associations between novel objects and locations in which objects have not appeared previously.

It is possible that enhanced fast gamma coupling in the hippocampal network during exploration of novel object–place associations coordinates ensembles of place cells that encode information about the location and the objects. If so, then place cell spiking should be more strongly modulated by fast gamma rhythms during exploration of novel object–place associations. To investigate this possibility, phase-locking of CA3 and CA1 place cell spikes to slow and fast gamma in CA1 was assessed in the subset of place cells that coded locations close to novel or familiar object–place pairings (*n* = 98 CA1 cells and 33 CA3 cells; Fig. 5). In each novel or familiar condition, place cells with place fields near either object were identified (see Materials and Methods, Place cell phase-locking). The phase-locking of place cells changed differentially depending on the novelty condition, place cell type (ie, field close to familiar or novel object–place pairing), and gamma type (Fig. 5; novelty condition x place cell type × gamma type interaction: *F*_(1,398)_ = 4.0, *p* = 0.05^g^, generalized linear mixed models). In the NO+NL condition, a significant interaction was found between gamma type and place cell type on the mean vector length of gamma phase distributions (Fig. 5*A,E*; *F*_(1,110)_ = 4.8, *p* = 0.03^g^, generalized linear mixed models, *n* = 16 cells with fields close to novel Object C and *n* = 41 cells with fields close to familiar Object A), indicating that phase-locking to fast gamma was more strongly affected by the presence of novelty than was phase-locking to slow gamma. Accordingly, spikes of cells near the novel object were significantly more phase-locked to fast gamma than were spikes of cells near the familiar object (Fig. 5*A,E*; *p* = 0.008^g^, *post hoc* for general linear mixed models). Analogous phase-locking effects were not observed for slow gamma (*p* = 0.9^g^, *post hoc* for general linear mixed models), nor were significant effects obtained across the other novelty conditions (Fig. 5*B*,*C,E*; NL: *F*_(1,100)_ = 4.1, *p* = 0.05^g^ for gamma type × place cell type interaction, *p* = 0.2^g^, *post hoc* for fast gamma, *p* = 0.4^g^, *post hoc* for slow gamma; NO: *F*_(1,132)_ = 0.4, *p* = 0.5^g^ for gamma type × place cell type interaction, *p* = 0.05^g^, *post hoc* for fast gamma, *p* = 0.2^g^, *post hoc* for slow gamma; generalized linear mixed models). These findings suggest that fast gamma rhythms may coordinate ensembles of place cells that signal object novelty and code spatial information for locations where objects were not previously found.

The phase-locking of place cell spikes to fast gamma rhythms during encoding of novel object–place pairings may also be associated with differences in firing rates. CA1 place cell in-field firing rates were significantly different, depending on which type of gamma was present and whether a cell’s field was located near a novel or familiar object–place pairing (Fig. 6A–*D*; place cell type × gamma type interaction: *F*_(1,146)_ = 4.6, *p* = 0.04^h^, generalized linear mixed models, *n* = 33 place cells with fields close to novel objects and *n* = 44 place cells with fields close to familiar objects). In the NO+NL condition, the in-field firing rates of CA1 place cells with fields near novel Object C, but not familiar Object A, were significantly higher during fast gamma periods than during slow gamma periods (Fig. 6*A*,*D*; place cell type × gamma type interaction: *F*_(1,56)_ = 4.5, *p* = 0.04^h^, generalized linear mixed models; cells with field near Object C, *p* = 0.04^h^; cells with field near Object A, *p* = 0.2^h^, sign test; *n* = 18 cells with fields near familiar Object A and n = 12 cells with fields near novel Object C). This result was not explained by effects of spiking on local fast gamma power because comparable findings were observed when place cell rate maps were constructed for slow and fast gamma episodes detected using non-local tetrodes (Fig. 6*E*; NO+NL: cells near Object C, *p* = 0.04^i^; cells near Object A, *p* = 0.5^i^; sign test). Similar findings were observed for object–place associations in the NL condition (Fig. 6*B*,*D,E*; gamma detected using local EEG: cell type × gamma type interaction: *F*_(1,38)_ = 33.5, *p* < 0.001^h^, generalized linear mixed models; place cells with field near Object A′, *p* = 0.008^h^; cells with field near Object A, *p* = 0.09^h^, sign test; n = 13 cells with fields close to A and n = 8 cells with fields close to A′) but not in the NO condition (Fig. 6*C*,*D*; cell type × gamma type interaction: *F*_(1,48)_ = 1.3, *p* = 0.3^h^; main effect of cell type: *F*_(1,48)_ = 0.02, *p* = 0.9^h^; main effect of gamma type: *F*_(1,48)_ = 0.005, *p* = 0.9^h^; generalized linear mixed models; *n* = 13 cells with fields close to A and *n* = 13 cells with fields close to B).

The above results suggest that the timing of fast gamma is optimally suited for encoding of novel object–place associations and that fast gamma may bring about increases in CA1 place cell firing rates during novelty exploration. Still, gamma power in CA1 is largest when theta is present (Csicsvari et al., 2003), raising the possibility that these effects simply reflect changes in theta power. However, no significant changes in theta power^j^ or theta phase synchrony^k^ between CA3 and CA1 were observed during exploration of novel-object place associations (Fig. 7*A-F*). Significant results were also not obtained when a stricter criterion for defining object exploration was used (see Materials and Methods; Table 1 ^j^). Still, the effects of novelty on theta power were rather variable (Fig. 7*D,E*), and thus it is possible that novelty-associated changes in theta power would achieve statistical significance in a larger dataset with more statistical power.

Next, effects of novelty on theta modulation of place cell spikes were assessed. There was a significant novelty condition x place cell type (ie, cells with fields close to familiar or novel object–place pairings) interaction effect on mean vector length of theta phase distributions of place cell spikes (Fig. 7*G*; *F*_(2,195)_ = 3.1, *p* = 0.05^l^, two-way ANOVA). In the NO+NL condition, spikes of place cells with fields near the novel object were significantly more phase-locked to theta than were spikes of cells with fields near the familiar object (*t*_(55)_ = 2.2, *p* = 0.03^l^, Student’s *t* test). Analogous phase-locking effects were not observed across the other novelty conditions (NL: *t*_(50)_ = 0.8, *p* = 0.4^l^; NO: *t*_(66)_ = 1.0, *p* = 0.3^l^, Student’s *t* test). Taken together with the fast gamma results reported above (Fig. 5*A*), this finding suggests that entrainment of place cell spikes by theta and fast gamma is enhanced during encoding of novel object–place associations.

## Discussion

These results suggest that fast gamma may coordinate neuronal activity in the hippocampal network during encoding of novel object–place associations. When novelty was defined by a new object in a location where an object had not been presented previously, several significant results were observed that were specific to fast but not slow gamma rhythms. First, there was a significant increase in CA1 fast gamma power during novel object exploration relative to familiar object exploration. Additionally, novelty exploration enhanced fast gamma phase synchrony between CA3 and CA1 relative to slow gamma CA3–CA1 phase synchrony, suggesting that fast gamma may couple CA3 and CA1 during encoding of novel object–place pairings. Also, place cells that fired near locations of new objects were more strongly modulated by fast gamma phase and theta phase than were place cells that fired near locations of familiar objects. This suggests that fast gamma, together with theta, organizes place cell spiking activity during encoding of novel object–place associations. In support of this idea, place cell firing rates increased selectively during fast, but not slow, gamma episodes as rats explored novel, but not familiar, object–place pairings.

When novelty involved only a location change (NL), the only significant effects that were observed were increases in fast gamma power during exploration of the novel object–place pairing and place cell spiking near the novel object–place pairing during fast gamma periods. Thus, it is possible that fast gamma also enhanced encoding of novel object–location pairings when novelty only entailed a change in object location. However, this type of novelty was likely not as striking as novelty involving both object identity and location changes, considering that significant behavioral effects of novelty were not observed in the NL condition (Fig. 1*C*). It is possible that the NL condition produced other fast gamma effects that were too small to be detected.

No fast or slow gamma-related effects were observed when a familiar object was replaced by a novel object in the same location (NO). It is possible that the saliency of the novel experience is relatively low in this type of paradigm, in which only the object identity changes, compared to a paradigm in which novel objects are presented in changed locations (eg, NO+NL in the present study). In accord with this assumption, animals did not explore novel objects significantly more than familiar objects in the NO condition in the present study (Fig. 1*C*). In this type of paradigm, animals may recall the general experience of encountering an object previously in the same location, rather than simply responding to the novelty of the object. An earlier study reported increased slow gamma measures when animals explored novel objects in locations where other objects had been presented previously (Trimper et al., 2014). Such increases in slow gamma may reflect retrieval of a memory of previously encountering objects in the same location, considering that CA3 is thought to play a key role in memory retrieval (Sutherland et al., 1983; Brun et al., 2002; Steffenach et al., 2002) and slow gamma is thought to be generated by CA3 (Colgin et al., 2009; Schomburg et al., 2014). This explanation may also apply to another report of increased slow gamma in animals exploring a novel W-maze (Kemere et al., 2013). The animals had been trained on a similar W-maze previously and thus may have been retrieving their memory of the general task, in addition to responding to novel stimuli in the novel maze, considering that increases in both slow and fast gamma power were observed in the novel maze (Kemere et al., 2013). In any case, the role of slow gamma in spatial memory processes remains an interesting question for future study.

With regard to fast gamma, the effects observed during the NO+NL condition are consistent with the notion that fast gamma plays a role in encoding of novel experiences. Previous studies have suggested that fast gamma is important for transmitting positional information from MEC to the hippocampus. Place cells in area CA1 preferentially code “place-based” representations of space during fast gamma (Cabral et al., 2014), and ensembles of CA1 place cells more closely represent an animal’s location in real-time during fast gamma (Zheng et al., 2016). Such communication about current spatial experience during fast gamma may complement a broader role of fast gamma in encoding memories of novel experiences.

The hypothesis that fast gamma rhythms are important for novelty encoding is also supported by results from earlier studies. Enhanced fast gamma power has been observed in area CA1 in rats during exploration of a novel maze (Kemere et al., 2013). A study in monkeys revealed an increase in coherence between hippocampal spikes and fast gamma rhythms during successful encoding of novel images (Jutras et al., 2009). In humans, an increase in higher frequency, but not lower frequency, gamma was observed in the hippocampus during successful encoding of words in a free recall task (Sederberg et al., 2007). The present results may similarly indicate formation of a novel associative memory during enhanced fast gamma activity, when transmission of novel sensory information to the hippocampus from MEC is likely to be strongest.

We did not find evidence for increases in Beta2 oscillations (∼25–30 Hz; Fig. 2*A-C*). These oscillations overlap in frequency with slow gamma and have been reported to increase in mice exploring novel environments (Berke et al., 2008; França et al., 2014). Such novelty-induced increases in Beta2 oscillations have not been reported yet in rats, and it is possible that effects of novelty on hippocampal oscillations differ between rats and mice.

Another recent study reported increased slow gamma phase-locking of place cell spikes in rats exploring a novel environment for the first time (Kitanishi et al., 2015). It is unclear why increased slow gamma phase-locking occurred in a novel environment in the study by Kitanishi et al. (2015), whereas increased fast gamma phase-locking occurred during presentation of novel object–place pairings in a familiar environment in the present study. Additional investigations are required to determine why slow gamma plays a role in encoding completely novel environments but not novel object–place pairings in a familiar setting.

A surprising finding in the present study was that fast gamma coupling between CA3 and CA1 was stronger than slow gamma coupling during exploration of novel object–place pairs (Fig. 4*A*). This is in contrast to other studies reporting that CA3 and CA1 are coupled by slow gamma during exploration of familiar environments (Colgin et al., 2009). It is possible that novelty induces neuromodulatory changes that allow fast gamma oscillators in CA3 and CA1 to become coupled. A plausible candidate for such a neuromodulator is acetylcholine. Hippocampal acetylcholine levels have been shown to increase in response to novel stimuli (Acquas et al., 1996). Also, the muscarinic receptor antagonist scopolamine, a drug that blocks memory encoding, suppressed fast gamma rhythms in MEC of behaving rats (Newman et al., 2013), suggesting that acetylcholine may enhance production of fast gamma rhythms in the hippocampus. The coupling of CA3 and CA1 by fast gamma during novelty may be necessary to ensure that memories of new experiences are stored in CA3–CA1 synapses.

The present study also found that place cell spiking was higher during fast gamma than during slow gamma when rats explored novel object–place pairings (Fig. 6). This effect could also involve increased acetylcholine release during novelty and subsequent enhancement of fast gamma, considering that acetylcholine increases place cell firing rates (Brazhnik et al., 2003). Another recent study investigated CA1 place cell firing rates during exploration of novel object–place pairings and found that mean firing rates were higher during novelty sessions compared with familiarity sessions (Larkin et al., 2014). However, the increased firing rates were not limited to periods when animals explored novel object–place pairings. The present results extend these findings by investigating place cell firing during slow and fast gamma. In the present study, in-field firing rates were increased during fast gamma periods relative to slow gamma periods when rats explored novel but not familiar object–place pairings in the NO+NL and NL conditions (Fig. 6*D*).

Novelty exploration was also associated with increases in fast gamma phase-locking of CA3 and CA1 place cell spikes (Fig. 5). Fast gamma phase-locked spiking across fast gamma cycles within a theta cycle resembles a stimulation paradigm (ie, “theta burst stimulation”) that is used to induce synaptic changes thought to underlie memory formation (Larson and Lynch, 1986; Larson et al., 1986). Thus, during encoding of novel experiences, changes in place cell spiking during fast gamma may augment memory encoding-enhancing effects of acetylcholine (Hasselmo, 2006) by directly promoting increases in synaptic strength.

## Synthesis

The decision was a result of the Reviewing Editor Kathryn Jeffery and the peer reviewers coming together and discussing their recommendations until a consensus was reached. A fact-based synthesis statement explaining their decision and outlining what is needed to prepare a revision is listed below. The following reviewers agreed to reveal their identity: James Knierim, James Ainge
